# From plant hormones to human health: progress and challenges in strigolactone biosynthetic structural diversity and antitumor effects

**DOI:** 10.3389/fpls.2026.1790032

**Published:** 2026-05-14

**Authors:** Xiaoxu Li, Suxing Tuo, Guoxin Chen, Changbin Niu, Yiqiong Liang, Daozhu Dong, Wei Liu, Kejun Zhong, Zhiyuan Li, Jianfeng Zhang, Wei Luo, Bo Kong

**Affiliations:** 1 Key Laboratory of Biosynthesis and Biomanufacturing in Model Plants (Beijing Life Science Academy), Ministry of Industry and Information Technology, Beijing, China; 2 Technology Center, China Tobacco Hunan Industrial Co., Ltd., Changsha, China; 3 Tobacco Research Institute, Chinese Academy of Agricultural Sciences, Qingdao, China; 4 Hunan Research Center of the Basic Discipline for Cell Signaling, Hunan Provincial Key Laboratory of Plant Functional Genomics and Developmental Regulation, College of Biology, Hunan University, Changsha, China; 5 Hunan Key Laboratory of Diagnostic and Therapeutic Drug Research for Chronic Diseases, Xiangya School of Pharmaceutical Sciences, Central South University, Changsha, China

**Keywords:** anti-inflammatory, antitumor, autophagy inhibition, biosynthesis, ferroptosis, P450, strigolactones

## Abstract

Strigolactones are plant growth regulatory compounds produced by plants through the breakdown of carotenoid pigments. The continued identification of key strigolactone biosynthetic enzymes and pathway branches, along with increasing evidence that synthetic strigolactone compounds have diverse biological activities, has led to the concept that strigolactones not only serve a dual role as plant hormones but also represent a class of potential medicinal scaffolds. Recent research summarizes the key components of strigolactone biosynthesis, highlights the conserved backbone and the key intermediate MeCLA generated through methylation and oxidation steps. Furthermore, the role of cytochrome P450 enzymes at the final stages of cyclization and oxidative modifications is highlighted to provide a molecular basis for the ability to generate canonical and noncanonical strigolactones simultaneously. This biosynthetic diversity is pharmacologically relevant since enzyme generated differences in scaffold architecture and oxidation pattern expand the strigolactone chemical space available for structure guided analog design and usage. In the field of biomedicine, strigolactone analogues have anticancer potential, which are able to inhibit the proliferation of tumor cells and modulate the process of angiogenesis, and most importantly, represent a new class of late-stage autophagy and mitophagy inhibitors that prevent the fusion of autophagosomes with lysosomes and enhance the efficacy of antitumor therapy. Strigolactones possess additional therapeutic activity, strigolactones have antioxidant and anti-inflammatory properties. Furthermore, there are several key barriers to strigolactone based drug development that have been identified, including chemical instability, inadequate *in vivo* testing for safety and metabolism profiles, and the absence of defined mammalian targets. Current evidence also indicates that the potential is more strongly supported for synthetic strigolactone inspired analogs than for canonical natural strigolactones. The future of strigolactones as potential drug candidates lies in their optimization through structure activity relationships and systems pharmacology validation for their target identification.

## 1 Introduction

Strigolactones (SLs) are a class of sesquiterpene lactone-type signaling molecules originating from plants, first identified as compounds secreted by plant roots. The term strigolactone traces back to Strigol named in association with the parasitic plant Striga (*Striga asiatica*) (Cook et al., 1966). SLs were subsequently established as a distinct group of phytohormones that regulate diverse developmental programs, including suppression of shoot branching, promotion of primary root and root hair growth, and control of leaf senescence and seed germination (Gómez-Roldán et al., 2008; Umehara et al., 2008). SLs are also, depending on the method in which they are generated from a plant, either mediated through processes occurring inside a plant or by being released from the root into the surrounding soil, in addition to their role as cues that promote arbuscular mycorrhizal symbiosis and stimulate the germination of seed bearing parasitic plants. SLs have received considerable attention in plant biology and agriculture, and as such, the focus on the potential biomedical uses continues to expand as the information related to SLs continues to be increased. There has been recent interest in the pharmacological properties of SL and many SL related analogs, with literature demonstrating broad anti-inflammatory, anticancer and antioxidant activities (Dell’Oste et al., 2021; Prandi et al., 2021; Pyrzanowska-Banasiak et al., 2023). The purpose of this review is to summarize recent advances in SLs biosynthesis and to summarize advances with respect to the effects of SLs and mechanisms of action in relation to the disease processes that occur in cancer and inflammation and to define future directions for SLs research.

## 2 From carotenoids to phytohormones: the strigolactone biosynthetic network

**Figure 1:**
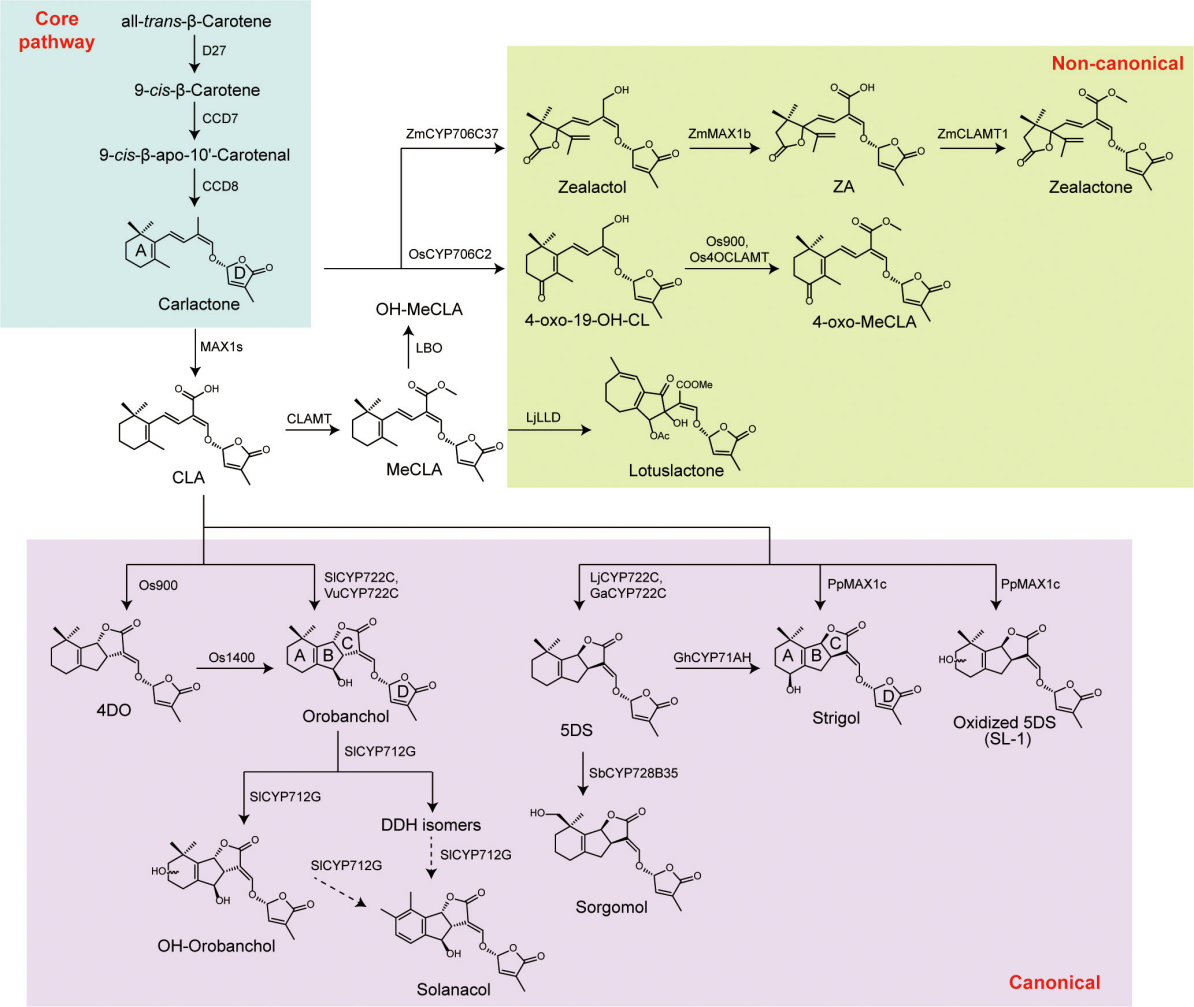
Overview of the strigolactone biosynthetic network and pathway diversification. The shared core pathway is shown in cyan, canonical strigolactone branches in violet, and non-canonical branches in green. This color coding highlights the conserved biosynthetic backbone and the lineage specific diversification steps that generate distinct SL chemotypes.

The synthesis of SLs is derived from a common carotenoid precursor via plants specific tailoring of the core precursors to produce a vast diversity. The studies completed recently supports a model where the final oxidative modifications, which is the final step prior to SLs synthesis, dominate the identity and concentration of SLs, and suggest that different products produced from one species are likely modular rather than strictly linear in terms of their production (**Figure 1**).

### 2.1 Canonical biosynthetic pathway

Strigolactones are carotenoid derived metabolites whose biosynthesis begins with carotenoid substrates such as β-carotene (Al-Babili and Bouwmeester, 2015; Mashiguchi et al., 2021; Zhou et al., 2025). In the canonical route, the carotenoid isomerase D27 and the cleavage enzymes CCD7 and CCD8 act sequentially to convert all trans-β-carotene into the initial SL precursor, carlactone (CL) (Lin et al., 2009; Booker et al., 2004; Alder et al., 2012). CL possesses a characteristic ABC-ring framework together with an unclosed unsaturated side chain and serves as a common precursor for all SLs. These upstream steps constitute a conserved core and are widely retained across plant kingdom (Mashiguchi et al., 2021). Downstream, the cytochrome P450 monooxygenase family CYP711A (MAX1 in *Arabidopsis*) oxidizes CL to carlactonoic acid (CLA), and methyltransferase activity generates the methylated intermediate methyl carlactonoate (MeCLA) (Mashiguchi et al., 2022). Building on these shared intermediates, plants introduce species and lineage-specific tailoring reactions that yield structurally diverse SLs. In some species, additional P450-catalyzed cyclization reactions convert CLA or its derivatives into defined SLs such as 5-deoxystrigol or strigol (Wakabayashi et al., 2019). P450 enzymes including CYP711A and CYP722C cooperate in these terminal steps, and members of these families in cotton and sorghum can directly convert CLA into SL structures such as orobanchol and 5-deoxystrigol (Wakabayashi et al., 2019; Niu et al., 2025). Additional enzymes have also been implicated in late stage modification. In *Arabidopsis*, LBO oxidizes MeCLA to 1’-hydroxy-MeCLA and can feed back to regenerate CLA, whereas in *lotus* a newly described dioxygenase (LjLLD) catalyzes formation of a noncanonical SL product, lotuslactone (Mashiguchi et al., 2022).

### 2.2 P450-driven structural diversification

Structure and composition of the SLs analyzed are clearly diverse among plant species with different biosynthetic routes to generate SLs and various enzymes capable of making SLs from other biosynthetic/substrate materials. Evidences show that cytochrome P450s are likely to be major determinants for the structural diversity of SLs (Mashiguchi et al., 2021; Niu et al., 2025). As an example of the way to study and discover additional SL related P450s, gene co-expression analysis, gene editing techniques, and functional confirmation can all be done using genetic material from organisms that are heterologously expressed in yeast, tobacco, or other systems (Wakabayashi et al., 2019; Li et al., 2024). It was reported that the Sugimoto group identified CYP722C family members found in legumes and other plants that were capable of catalyzing the conversion of CLA to specific groups of SLs (Wakabayashi et al., 2019). This result demonstrated that the terminal branching observed was not limited to the MAX1 pathway and suggested an additional route for forming SL products that did not include CYP711A. Thus, CYP711A, CYP722C, and the grass-specific CYP706 P450s are grouped based on their structures and events leading to the formation of SL products (Mashiguchi et al., 2021; Niu et al., 2025). The emergence and diversification of these P450 enzymes are therefore regarded as major forces shaping lineage specific SL repertoires (Niu et al., 2025).

### 2.3 New discoveries in noncanonical branches

Research on rice (*Oryza sativa*) has shown that while the majority of existing studies focus on canonical SLs which are produced via the most mainstream pathway and are currently considered primary and secondary roots. However, in addition to these pathways there are additional “noncanonical” pathways that also contribute to the biosynthesis of SL products (Li et al., 2024). The previous study identified the following components of the rice SL biosynthetic pathway, the biosynthetic enzyme, OsCYP706C2, and two methyl transferases, mediating the adjacent reaction from carlactone to 4-Oxo-MeCLA (Li et al., 2024). Cross-species co-expression analysis identified these three proteins as essential to producing this noncanonical SL. Furthermore, heterologous expression of OsCYP706C2 and a methyl transfer proposal indicated that the enzyme uses carlactone as a substrate to convert it into 4-Oxo-MeCLA in conjunction with a methyl transferase (Li et al., 2024). The *oscyp706c2* mutant has not been observed to present any drastic alterations in tillering phenotype, indicating that the OsCYP706C2 mediated noncanonical SL biosynthetic pathway does not play a major role in determining the shoot architectural characteristics of rice, however, the *oscyp706c2* mutant does exhibit delayed mycorrhizal colonization and altered root architecture suggesting that the OsCYP706C2 mediated branch of the pathway appears more closely to relate to symbiotic relationships and the physiology of roots than to shoot branching promotion or signaling for parasitic plants (Li et al., 2024). Additionally, these findings are consistent with the hypothesis that although this is a distinct SL biosynthetic pathway to the canonical SL biosynthetic pathway, both of these pathways serve unique ecological functions.

Meanwhile, researchers found a second group in corn through screening of germplasm (Li et al., 2023). That study identified a line of corn whose roots produce a different composition of secreted strigolactones than were noted in the majority of corn lines and that was resistant to witchweed (*Striga*). This study also demonstrated that the corn resistance line produces two strigolactones (zealactol and zealactonoic acid). These two SLs demonstrated weak activity in stimulating witchweed germination compared to the previously documented active strigolactone zealactone. Mechanistic studies demonstrated that the enzyme, ZmCYP706C37, a cytochrome P450 oxidase, catalyzes several steps of oxidation of metabolites resulting in conversion of these intermediates into zealactone (Li et al., 2023). Further analyses of resistant lines demonstrated that due to the reduced activity or the loss of CYP706C37 from the biosynthetic pathway for the conversion of metabolites into zealactone, the flow of biosynthetic products is diverted toward the production of zealactol and zealactonoic acid and as a result, the strength of the germination signal is reduced. Furthermore, ZmMAX1b, ZmCYP711A, and ZmCLAMT1, a putative CLA methyltransferase, also contribute to the total SL phenotype of maize by participating along with ZmCYP706C37 in changing the SL composition profile (Li et al., 2023). Additionally, the changed compositions of SLs produced by these different lines will be less likely to be recognized by parasitic plants leading to reduced infection rates (Li et al., 2023). Together, the two studies provide additional insight into the SL biosynthetic pathway in maize and provide breeding strategies for altering the profile of strigolactones produced based on manipulating the activity of P450 and their downstream products to minimize parasitic stimulation (Mashiguchi et al., 2021; Li et al., 2023). In conclusion, recent studies of rice and maize provide evidence of the variability and complexity of SL biosynthesis, which can result from combined actions and interactions of different types of P450 and other enzymes.

### 2.4 From biosynthetic diversification to pharmacological opportunity

Notably, the biosynthetic diversification summarized above is not only relevant to plant adaptation, but also provide the chemical space available for biomedical exploration. Although the direct target of most SLs remain incompletely defined, these biosynthetic variants provide a useful framework for structure activity relationship (SAR) analysis, because even subtle changes in the SL scaffold may influence chemical stability, cellular uptake, and biological responses in mammalian systems. In this level, plant pathway diversity may offer a blueprint for prioritizing natural SL inspired chemotypes and for designing more stable and potent analogs for applications.

## 3 Antitumor activity and mechanisms: broad antitumor potential

**Figure 2:**
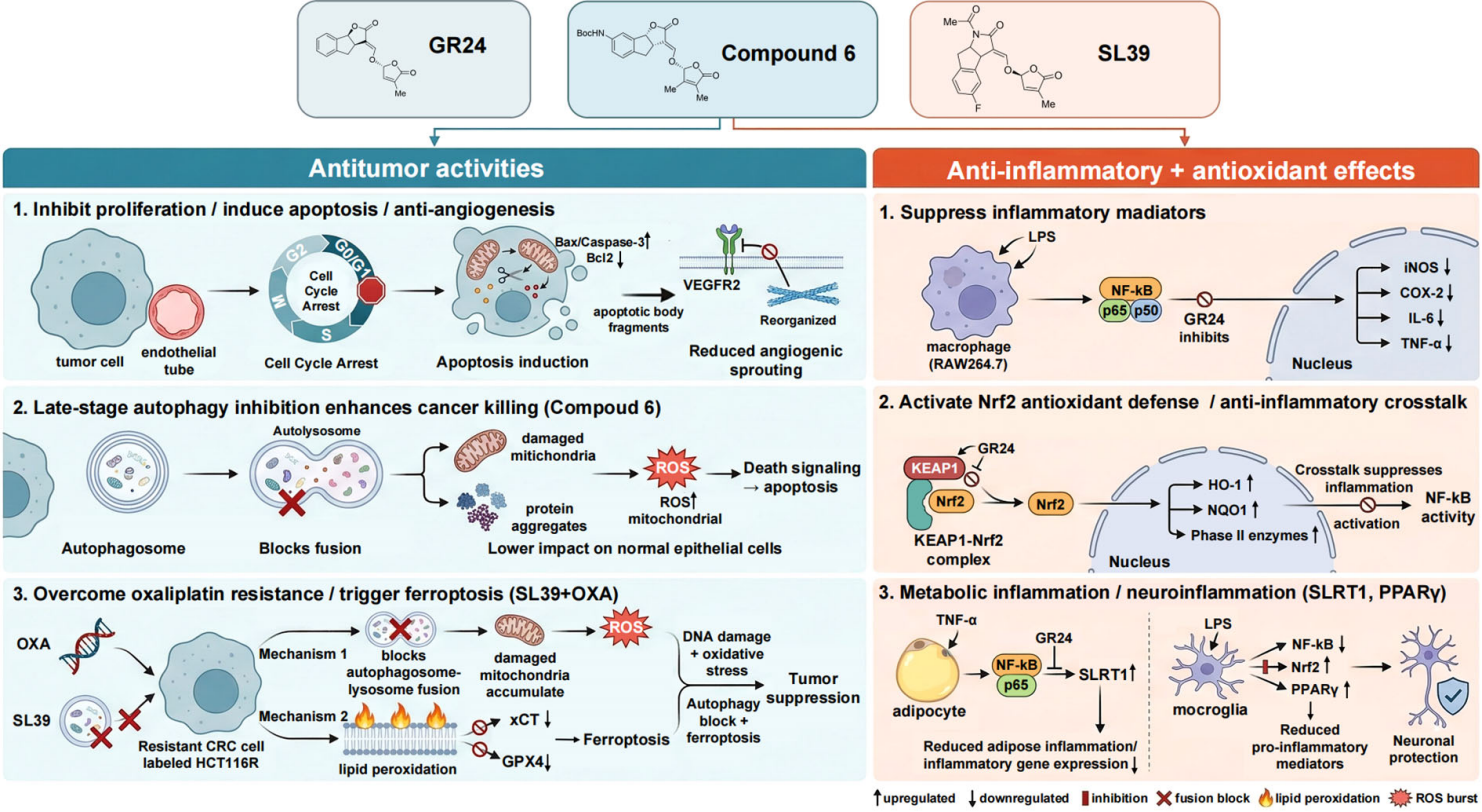
Proposed model summarizing experimentally supported and putative mechanisms of strigolactone analog mediated antitumor and anti-inflammatory activities. The figure highlights major pathway-level effects using concise labels. Notably, the direct protein targets of strigolactones remain undefined.

Under this biosynthetic background, the biomedical reports can be viewed as testing which regions of SL chemical space are compatible with bioactivity. Most compounds examined to date are synthetic analogs rather than endogenous plant SLs, but their designs are rooted in the conserved SL scaffold and in modifications inspired by biosynthetic diversification. Anti-cancer agents utilizing the phenolic compound SL, have progressively become known as a diverse groups of chemical compounds that effectively inhibit the growth of malignant cells, stimulate cellular apoptosis, and sometimes, offer potential therapeutic options for disrupting the establishment of an optimal blood supply to tumors through inhibiting angiogenesis. A recurring mechanistic theme is that selected derivatives weaken stress adaptation capacity, thereby enhancing therapeutic pressure, reducing drug tolerance, and creating conditions that favor apoptosis or ferroptosis, which together supports their potential in combination oriented cancer treatment (**Figure 2**). Notably, the most convincing evidence currently comes from synthetic SLs inspired analogs, suggesting that mammalian bioactivity may depend more on a pharmacologically optimized SLs scaffold than on the native architecture.

### 3.1 Inhibition of tumor-cell growth and induction of cell death

An increasing number of studies indicate that SL related molecules can suppress proliferation across multiple tumor cell types and selectively induce apoptosis in cancer cells. Using the synthetic SL analog GR24 as an example, (±)-GR24 shows antitumor activity in various cell lines, including inhibition of cancer-cell proliferation, induction of cell-cycle arrest, and promotion of apoptosis (Pollock et al., 2014; Mayzlish-Gati et al., 2015; Prandi et al., 2021; Pyrzanowska-Banasiak et al., 2023). Carrillo and colleagues further reported that GR24 can markedly inhibit tumor angiogenesis, an effect linked to interference with VEGFR2 signaling and to cytoskeletal reorganization (Carrillo et al., 2019). Given that angiogenesis is essential for the development of tumors and the subsequent invasion and dissemination of these tumors to distant sites in the body, the anti-angiogenic effects exhibited by SLs may therefore provide greater anti-tumor potential for use in the therapeutic treatment of cancer (Carrillo et al., 2019). In addition, evidence exists that combinations of SLs and other anticancer agents are effective against many forms of aggressive neoplasms such as glioblastoma (GBM). These reports show that two novel SL analogues not only demonstrated the ability to upregulate both the pro-apoptotic protein Bax and the pro-apoptotic protease Caspase 3, but also downregulated the anti-apoptotic protein Bcl-2, thereby inducing the activation of apoptotic pathways, inhibiting GBM cell proliferation, and inducing GBM cell cycle arrest. Collectively, these actions resulted in a dramatic reduction in GBM cellular viability, suggesting that these newly synthesized SLs will serve as leads in the development of efficacious anticancer agents for the treatment of glioblastoma (Antika et al., 2022). Thus, together these initial findings provide compelling evidence for the possible broad antitumor activity.

### 3.2 Enhancing anticancer efficacy through autophagy inhibition

Cancer cells often utilize elevated levels of autophagic activity as a means to survive in environments that are hostile and stressed by chemotherapy drugs. Therefore, inhibiting this process could potentially improve the effectiveness of treatment and, thus far, only a few effective and selective inhibitors of autophagy have been developed (Yang et al., 2022; Pyrzanowska-Banasiak et al., 2023). Notably, SL related molecules have recently been found to exhibit selective autophagy-inhibitory activity. The previous report designed and synthesized a series of SL analogs and, through screening, identified an optically pure derivative (Compound 6) that displayed potent and selective cytotoxicity toward colorectal cancer cells while exerting minimal effects on normal colorectal epithelial cells (Yang et al., 2022; Pyrzanowska-Banasiak et al., 2023). Structurally, Compound 6 appears to retain the characteristic butenolide D-ring/enol-ether pharmacophore of strigolactones while incorporating a rigid and medicinally optimized scaffold (**Figure 2**). In a nude-mouse subcutaneous xenograft model, Compound 6 dose-dependently inhibited HCT116 tumor growth without obvious systemic toxicity (Yang et al., 2022). Mechanistically, Compound 6 induced apoptosis and arrested the cell cycle at G0/G1 in colorectal cancer cells (Yang et al., 2022). Significantly, Compound 6 selectively inhibited late-stage autophagy (LSA) in HCT116 cells by increasing autophagic flux but blocking the fusion of autophagosomes and lysosomes (Yang et al., 2022). In doing so, Compound 6 disrupted a critical step in the fusion process, causing the accumulation of toxic metabolites and damaged organelles, which led to the activation of cell death signals. At the same time, normal cells, which typically have lower autophagic basal levels and have less sensitivity to death signals, were less affected than HCT116 cells (Yang et al., 2022; Pyrzanowska-Banasiak et al., 2023). This research presents evidence that one optically pure SL derivative can be utilized as a selective autophagic/mitophagy inhibitor with a novel mechanism, and supports that the inhibition of LSA is a promising approach for the treatment of colorectal cancer, and provide guidance for the design of cancer therapeutic agents (Yang et al., 2022).

### 3.3 Overcoming chemoresistance and inducing ferroptosis

Beyond direct tumor-cell killing, SL derived molecules have shown encouraging potential in reversing chemoresistance. Oxaliplatin (OXA) resistance remains a major clinical obstacle in colorectal cancer. The study reported a class of type-II strigolactams (designated SL 39) that strongly sensitized OXA-resistant colorectal cancer cells (HCT116R) (Liu et al., 2025). *In vitro*, SL 39 combined with OXA restored drug sensitivity in HCT116R cells; in nude-mouse xenografts, the combination therapy also effectively suppressed growth of resistant tumors (Liu et al., 2025). Mechanistically, SL 39 resembles Compound 6 in targeting LSA, it specifically blocks autophagosome-lysosome fusion in resistant HCT116R cells without substantially altering lysosomal pH (Liu et al., 2025; Yang et al., 2022), thereby leading to mitochondrial dysfunction and lethal accumulation of reactive oxygen species (ROS) (Liu et al., 2025). SL 39 also increases membrane lipid peroxidation and inhibits the cystine/glutamate antiporter xCT (SLC7A11) and glutathione peroxidase 4 (GPX4) (Liu et al., 2025), effectively converting protective autophagy into ferroptotic death (Liu et al., 2025). SL39 likely shifts protective autophagy toward ferroptosis by blocking LSA, increasing ROS, and weakening xCT/GPX4-dependent detoxification of lipid peroxides. Besides, the iron metabolism is also likely involved, although the specific downstream events remain undefined. Because this approach bypasses classical apoptosis pathways, it may remain effective in tumors with apoptosis resistance, including those with high BCL-2 expression or p53 mutations. Moreover, SL39 shows low toxicity toward normal intestinal epithelial cells and is well tolerated in mice, suggesting a favorable therapeutic window. When combined with OXA, the regimen produces a coordinated effect in which OXA induces DNA damage and oxidative stress while SL39 prevents stress relief via autophagy and triggers ferroptosis, resulting in synergistic killing of resistant cells (Liu et al., 2025). This study therefore proposes a novel and potentially translatable metabolic intervention strategy for overcoming oxaliplatin resistance in advanced colorectal cancer and broadens the prospects for plant derived small molecules in precision oncology (Liu et al., 2025). Taken together, these studies suggest an emerging SAR framework for SLs derived anticancer agents. In mammalian systems, potent activity may not require the full canonical SL architecture. Instead, optimized SL inspired analogs such as Compound 6 and SL39 may retain stronger anticancer effects through improved chemical robustness and intracellular persistence.

## 4 Anti-inflammatory biological effects: dual regulation of inflammation and oxidative stress

Strigolactones and their mimetic molecules demonstrate clearly defined anti-inflammatory effects by inhibiting the synthesis of pro-inflammatory mediators and antagonizing typical pathways of inflammatory signal transduction and activating the antioxidant defense systems, which enhance the cytoprotective response to oxidative stress produced during the inflammatory response. *In vitro* and other disease related experiments have demonstrated that the actions of SLs converge on a dual mechanism of action which regulates both inflammation and oxidative stress (**Figure 2**). A number of recently identified signals have demonstrated that SL based chemotypes may also regulate metabolic inflammation and affect neuroinflammatory processes.

### 4.1 Suppression of inflammatory mediators and signaling pathways

Furthermore, while inflammation is generally a protective response to injury or infection, chronic or excessive inflammation can lead to tissue damage and disease states. SLs exhibit significant anti-inflammatory activity at the cellular level (Zheng et al., 2018; Pyrzanowska-Banasiak et al., 2023). In laboratory animals, GR24 (synthetic SL analogue) has reduced inflammatory mediator production and inhibited inflammatory signal transduction in a number of different experimental models. Studies have shown a significant reduction in LPS induced iNOS and COX-2 expression after treatment with GR24, and a decrease in the release of proinflammatory cytokines (i.e., IL-6 and TNF-α) from RAW264.7 macrophage cells isolated from mice (Tumer et al., 2018). GR24 inhibits the activation and nuclear translocation of several proinflammatory transcription factors, including NF-κB (Tumer et al., 2018), and decreases the accumulation of nuclear NF-κB p65 in adipocytes, which can subsequently lead TNF-α induced chronic low-grade inflammation (Modi et al., 2018; Pyrzanowska-Banasiak et al., 2023). While the effects of GR24 on these cellular systems support the idea that SLs may inhibit inflammatory responses by blocking or disrupting proinflammatory signal transduction pathways, GR24 has only produced minimal or no effects when tested in cells that have not been stimulated. Thus, it is likely that GR24 acts as a selective anti-inflammatory agent, and this finding may ultimately be useful for the identification and development of new anti-inflammatory pharmaceuticals.

### 4.2 Induction of antioxidant and cytoprotective mechanisms

GR24 has been reported to activate the Nrf2 pathway (Tumer et al., 2018), a master regulator of antioxidant defense that also protects against inflammation associated damage. Under basal conditions, Nrf2 is restrained by Keap1. Based on molecular docking, it was proposed a putative interaction in which GR24 might interfere with the Keap1-Nrf2 complex (Tumer et al., 2018). However, this remains a computational prediction rather than a direct biochemical validation of Keap1. In parallel, GR24 reduces LPS induced expression of several inflammatory genes, including iNOS, IL-1β, and COX-2, and suppresses nuclear accumulation of NF-κB (Tumer et al., 2018). Nrf2 activation not only enhances antioxidant capacity but also limits inflammatory cytokine production via pathway crosstalk (Zheng et al., 2018), in part because Nrf2 induced outputs can interfere with NF-κB activation and thereby dampen transcription of pro-inflammatory genes (Zheng et al., 2018). Thus, by engaging endogenous antioxidant defenses, SLs can directly reduce oxidative injury and indirectly curb inflammatory signaling, a dual mode of action that may be particularly relevant to chronic inflammatory disorders.

### 4.3 Effects on metabolic and neuroinflammation

Beyond general immune inflammation, SLs have also been examined in metabolic and neuroinflammatory contexts. In metabolic syndrome related studies, Modi et al. reported that GR24 alleviates high fat diet, induced adipose inflammation in mice, involving activation of SIRT1 and downregulation of adipogenic and inflammation related gene expression (Modi et al., 2018). GR24 reduces pro-inflammatory factors secreted by hypertrophic adipocytes, thereby mitigating obesity associated chronic inflammation (Modi et al., 2018). In the nervous system, SLs have shown anti-neuroinflammatory and neuroprotective activities, suggesting possible relevance to early Alzheimer’s disease associated processes. It was reported that an SL analog exerts multiple effects in LPS stimulated microglia, including suppression of pro-inflammatory mediator release via NF-κB, Nrf-2, and PPARγ signaling and reduction of LPS induced neuronal permeability changes (Kurt et al., 2020).

## 5 Outlook: flexible control of biosynthetic branching and challenges in drug translation

Recent progress has substantially advanced SL research. From the plant side, systematic dissection of SL biosynthetic complexity, especially the identification of new enzymes with P450s being particularly prominent, has clarified how structural diversity is generated (Li et al., 2024; Niu et al., 2025). These advances enrich fundamental understanding of phytohormone biosynthesis and support applications in agriculture (Li et al., 2023). At the same time, SL research is increasingly extending into biomedicine. Extensive *in vitro* and *in vivo* evidence indicates that SL analogs exhibit antitumor and anti-inflammatory activities through diverse mechanisms, including suppression of tumor angiogenesis (Carrillo et al., 2019), blockade of autophagy (Yang et al., 2022), induction of ferroptosis (Liu et al., 2025), inhibition of inflammatory pathways, and activation of antioxidant responses (Tumer et al., 2018; Zheng et al., 2018). Together, these developments strengthen the view that SLs can move from “plant hormones” toward “candidate therapeutics” (Dell’Oste et al., 2021; Prandi et al., 2021). Future studies might explicitly integrate plant enzymology with medicinal chemistry, using biosynthetic methods to prioritize scaffold classes, guide SAR analysis, and improve stability and target selectivity.

Nonetheless, several limitations and challenges remain. First, most biomedical studies of SLs still focus on pharmacodynamic evaluation and mechanistic exploration. Before clinical translation, druggability and safety require more rigorous assessment. The metabolism and potential toxicity of SL in animals is poorly characterized, despite that SL are important trace signaling molecules in plants. Second, SL may have low chemical/metabolic stability, this can limit their usefulness practically, for example GR24 can degrade in solution and is sensitive to pyrimidine nucleosides and thiol-containing biomolecules (Pyrzanowska-Banasiak et al., 2023). Thus, lead optimization will likely require structural modifications to enhance the stability/safety of SL’s while improving their pharmacokinetic profile. Finally, the direct mammalian protein targets of SLs remain unidentified or insufficiently validated. Although docking and pathway level analyses have suggested putative interactions, including with iNOS and Keap1, direct target engagement has not yet been established experimentally (Tumer et al., 2018; Pyrzanowska-Banasiak et al., 2023). The identification and confirmation of the functional mammalian targets of SL using pharmacological approaches and chemical biology will be essential for elucidating the mechanisms of action involved and thus guiding the rational design of new SL drugs.

## 6 Conclusion

To summarize, strigolactones are considered “small molecules with big signals” due to their importance in plant biology, and growing evidence suggests that they have cross domain functions in biomedicine as well. Research is focused on antitumor/inflammatory properties, however, growing evidence indicates that these compounds also have broader potential such as their antidiabetic abilities. As more researchers become interested in this area, more therapeutic functions and opportunities will arise for SLs. Future studies will focus on drug based development through deeper mechanistic understanding, including structural optimization, structure activity relationship studies and also *in vivo* assessment experiments. In particular, the progress will depend on distinguishing plant structural features from mammalian pharmacophore requirements, improving hydrolytic stability, and building a clearer SAR framework for synthetic SL inspired chemotypes. Collaborative efforts between disciplines might assist SLs in growing from plant roots to new medical chapters contributing to plant derived therapies for diseases such as cancer and inflammatory disorders.
